# Dendritic Cell Vaccines in Ovarian Cancer

**DOI:** 10.3389/fimmu.2020.613773

**Published:** 2021-01-25

**Authors:** Xi Zhang, Tianhui He, Yuan Li, Ling Chen, Hongyu Liu, Yu Wu, Hongyan Guo

**Affiliations:** ^1^ Department of OB/GYN, Peking University Third Hospital, Beijing, China; ^2^ Department of Neurosurgery, First Medical Center of Chinese PLA General Hospital, Beijing, China; ^3^ Department of Neurosurgery, Hainan Hospital of Chinese PLA General Hospital, Sanya, China

**Keywords:** dendritic cells (DCs), ovarian cancer (OC), immunotherapy, tumor microenvironment, dendritic cell vaccine

## Abstract

Ovarian cancer (OC) is one of the most lethal malignant gynecologic tumors, characterized by an uncertain presentation and poor outcomes. With or without neoadjuvant chemotherapy, surgery followed by platinum-based chemotherapy and maintenance therapy are the basis for the treatment of ovarian cancer patients, but the outcome is still highly restricted by their advanced stage when diagnosed and high recurrence rate after chemotherapy. To enhance the anti-tumor effect and postpone recurrence, anti-VEGF agents and PARP inhibitors are suggested as maintenance therapy, but the population that can benefit from these treatments is small. Based on the interactions of immune cells in the tumor microenvironment, immunotherapies are being explored for ovarian cancer treatment. Disappointingly, the immune checkpoint inhibitors show relatively low responses in ovarian cancer. As shown in several studies that have uncovered a relationship between DC infiltration and outcome in ovarian cancer patients, dendritic cell (DC)-based treatments might have a potential effect on ovarian cancer. In this review, we summarize the functions of dendritic cells (DCs) in the tumor microenvironment, as well as the responses and drawbacks of existing clinical studies to draw a comprehensive picture of DC vaccine treatment in ovarian cancer and to discuss the promising future of immune biomarkers.

## Introduction

Ovarian cancer is the most lethal gynecological cancer, with an overall 5-year-survival rate of 48% (US, reported in 2020). Nearly 75% of patients have no symptoms until an advanced stage, which leads to a 29% 5-year-survival (1). First-line treatments include surgery and platinum-based chemotherapy. Although primary treatments show remission effects, approximately 75% of patients suffer from recurrences, followed by eventual drug resistance state.

When confronted with recurrences, platinum-sensitive patients are recommended to accept platinum-based combined chemotherapy followed by targeted therapy according to the NCCN guidance. However, platinum-resistant recurrences have limited effective strategies to choose. The response rates to cytotoxic therapy [ex. topotecan, 20% (2); docetaxel, 22% (3)] and single agents targeted therapy [ex. bevacizumab, 20% (4)] are low, and furthermore, a combination of chemotherapy and bevacizumab increase the median overall survival by only 3.3 months, with no significant difference between the combination therapy and chemotherapy groups (5). Consequently, studies have focused on maintenance therapy to postpone any recurrence (6). Poly ADP-ribose polymerase (PARP) inhibitors, including olaparib, niraparib, and rucaparib, have manifested inspiring efficacy in maintenance therapy. Olaparib for those with BRCA1/2 mutations has increased the response rate to 36% (7), however, BRCA1/2 mutations exist in only 10% of ovarian cancer patients. Although niraparib and rucaparib extend their indications to those with homogenous repair deficiency (HRD) (8) as well as those with platinum-sensitive recurrent epithelial ovarian cancer (EOC) regardless of BRCA status (9), most patients still do not qualify. Emerging studies aim to elongate recurrence intervals for better survival, and immunotherapy is being considered.

The presence of tumor infiltration lymphocytes is related to a higher 5-year-survival rate (38% vs 4.5%) in ovarian cancer (10), which throws light on immunotherapy. However, the efficacy of immune checkpoint blockers, such as the anti-PD-1 agent pembrolizumab, depend on microsatellite instability-high or mismatch repair-deficient circumstances. The overall response rate to pembrolizumab among PD-L1^+^ advanced metastatic ovarian cancer patients is only 11.5% (11), and the percentage of PD-L1^+^ cases of high grade serous ovarian cancer is only 57.4%, and it is 0%–26.7% in other histologic subtypes of ovarian cancer (12). Moreover, adoptive T cell therapy is hindered by a low level of T cell infiltration, poor neoantigen presenting function and immunological tolerance epitopes (6), which suggests to enhance the process of antigen-presenting for amplifying anti-tumor effect.

In the tumor microenvironment, dendritic cells take and process tumor-associated antigens, then present them by MHCI/II molecules to activate T cells. With the aim of enhancing the process of antigen-presenting, DCs are regarded as promising target. The first clinical trial of dendritic cell (DC) vaccine started in 1996. Currently, more than 400 clinical trials of DC-based treatment for tumors have been registered in ClinicalTrials.gov. Up till June 2014, DC-based treatment in only four tumor types had reached phase III clinical trials, including melanoma, prostate cancer, malignant glioma and renal cell cancer (13). During 2014–2017, 43 peer-reviewed publications reported the outcomes of clinical trials on DC vaccines (14) in various cancers. In most clinical trials, some of the patients reached a stable state, and a lower percentage of patients reached a partial response or a complete response (according to RECIST guidelines). During 2017–2019, 34 peer-reviewed papers were published (15) suggesting more strategies to improve the response rate to DC vaccines.

Safety was confirmed in most clinical trials and the response rate to DC-based treatment gradually increased due to improved production strategies. Antigen loading, DC origination and induced maturation strategies are key steps to produce DC vaccines, which stand at the core stage of innovation. In 2010, Sipuleucel-T became the first DC vaccine approved by the FDA, for the treatment of metastatic prostate cancer. In ovarian cancer, various DC vaccines have been tested, showed an increment in progression-free survival (PFS) and overall survival (OS) (16, 17), which inspired further studies.

## Differentiation, Maturation, and Function of the Dendritic Cells

**Figure 1:**
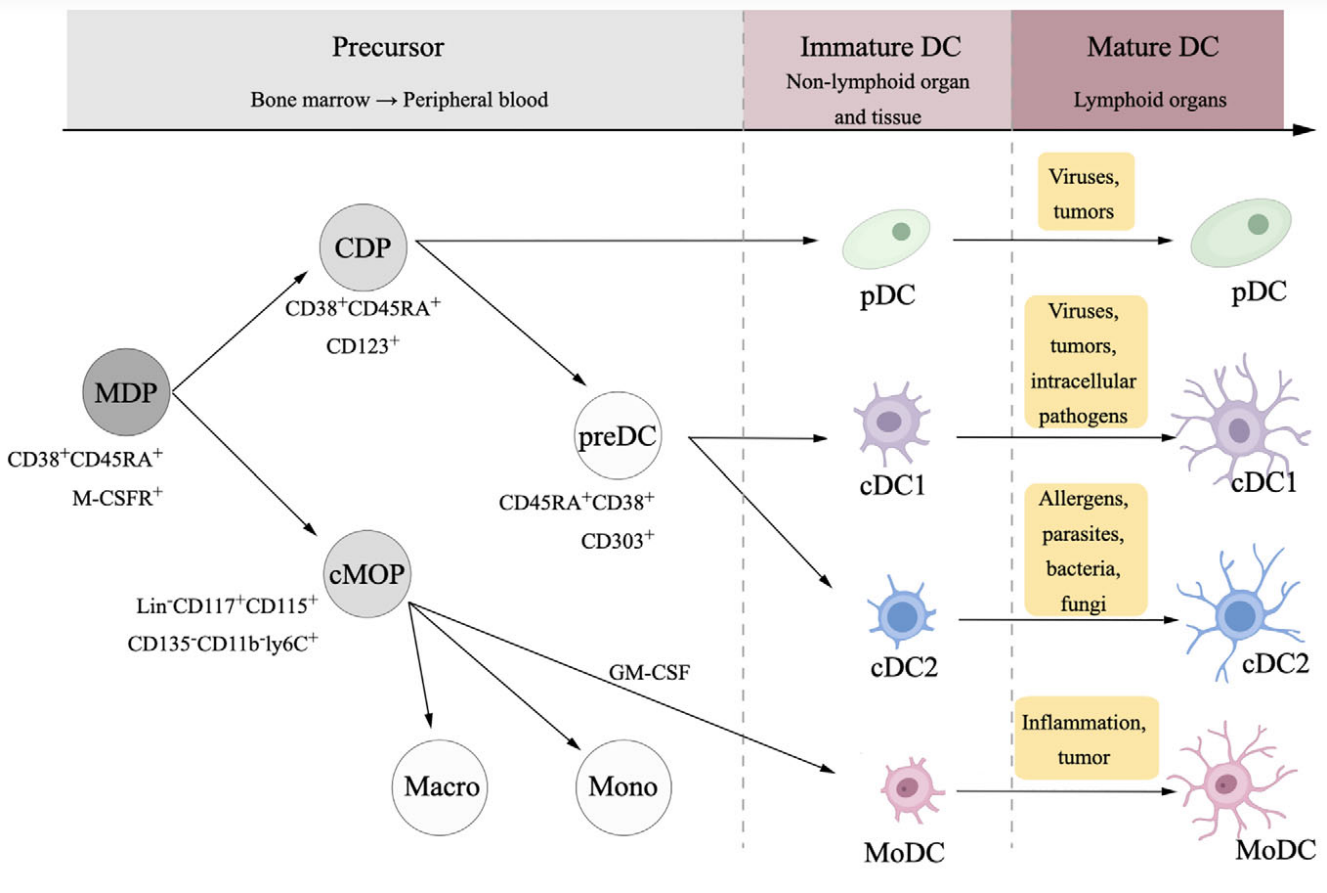
Differentiation and maturation of dendritic cell (DC). DCs and monocytes originate from a common ancestor, namely macrophage dendritic cell progenitor (MDP). MDPs differentiate to common monocyte progenitors (cMOPs) and common DC progenitors (CDPs). cMOPs generally differentiate into monocytes and macrophages, while in some situations with pro-inflammatory context, cMOPs could be stimulated by granulocyte-macrophage colony stimulating factor (GM-CSF) then differentiate to monocyte-derived dendritic cells (MoDCs). CDPs differentiate to two subsets, plasmacytoid DCs (pDCs), and myeloid dendritic DCs, which is usually called conventional DCs (cDCs).

DCs originate from CD34^+^ hemopoietic stem cells in the bone, differentiate to different subtypes in the peripheral blood and nonlymphoid organs and tissues, and mature in the lymphoid organs (18–21) (**Figure 1**). Immature DCs that express low levels of toll-like receptors (TLRs) MHC molecules, costimulatory molecules, as well as adhesion molecules stay outside of lymphoid tissues and have weak antigen-presenting functions. TLRs are the essential receptors among the sensors of pathogen-associated molecular patterns (PAMPs) and damage associated molecular patterns (DAMPs). PAMPs from bacteria, viruses, or parasites activate DCs to activate the innate immune response (22), which act as a general defense against infectious diseases; in tumors, DCs are activated in response to DAMPs from tumor cells through TLR signaling (23).

The migration of immature DC is induced by the chemokine receptor CCR7 and CCR8, which is up-regulated during the maturation process (24). Stimulated by antigens, immature DCs migrate toward chemokine ligands CCL19 and CCL21 into lymph nodes and gradually develop into a mature state, highly expressing MHC I molecules, MHC II molecules, costimulatory molecules and adhesion molecules (25), then mature DCs active CD4^+^T cells and CD8^+^T cells at the tumor site migrate to lymphoid organs to build immune memory.

As robust antigen-presenting cells, DCs exert a key influence in regulating the innate immune response and initiating adaptive immune responses. DCs have more vigorous capability to capture, process and present antigens than other APCs, such as B cells, mononuclear cells, and macrophages (26). DCs take parts in forming the first and second signals to activate T cells. In the process of activating specific T cells, the MHC-antigen peptide-TCR complexes act as the first signal, and the costimulatory factors on the membrane of APCs act as the second signal. Generally, DCs capture and process exogenous antigen peptides into antigen peptide/MHC II molecules in order to activate CD4^+^ helper T cells, and endogenous antigen into antigen peptide/MHC I molecules for CD8^+^ T cells (27, 28).

In tumors, different subtypes of DCs play divergent roles. Conventional dendritic cell type 1 (cDC1) and conventional dendritic cell type 2 (cDC2) are two subtypes of cDCs. cDC1 is the main DC subtype that activates CD8^+^ T cells through the antigen cross-presentation process (29), while cDC2 secretes IL-10, IL-12, IL-23, and TNF-β to stimulate the differentiation of the CD4^+^T cells (30). It is cDCs that mainly present tumor antigens and promote antitumor effects.

Plasmacytoid DCs (pDCs) may be involved in both tumor protective processes and tumor-suppressive processes. On one side, pDCs mainly secret type I IFN, which is essential antitumor cytokine (31). On the other side, pDCs induce immunosuppressive cells, leading to a poor outcome (25, 32). Due to potentially bidirectional effect, the role of pDCs may be dependent on the tumor microenvironment. pDC is the main subtype of DCs in the tumor sites of ovarian cancer (33), and the infiltration of pDCs in the ovarian cancer microenvironment has a negative association with the prognosis (34), but the pDC in response to TLR could release IFN-α, although such type of function is weaker than that in the peripheral blood (35). The involvement of DCs in antitumor effects may be disturbed in ovarian cancer, which indicates a potential benefit of DC vaccines.

Monocyte-derived dendritic cells (MoDCs) originate from monocytes in the peripheral blood. The differentiation of MoDCs is commonly induced by GM-CSF and IL-4, followed by the maturation of immature MoDCs stimulated by tumor-associated antigens and other agents (36). MoDCs mainly respond to inflammation in the mouse, but human MoDCs are mostly studied *in vitro*, and their function depends on the stimulatory signals in the culture.

## Dendritic Cell Dysfunction in the Tumor Microenvironment

Ovarian cancer lesions have a high degree of DC infiltration, but infiltrated DCs have low efficacy of antigen presentation due to DC tolerance, which is marked by downregulated expression of costimulatory molecules on the surface of DC cells (37), as well as having weaker antigen-presenting ability. DCs also act to assist tumor cells in some situations. In the tumor microenvironment, it has been confirmed that many aspects could induce dysfunction of DCs, as discussed below.

Immune checkpoint signaling may participate in DC dysfunction. The combination of programmed cell death protein 1 (PD-1) on T cells and programmed death-1 ligand (PD-L1) on tumor cells leads to the programmed death of the T cells. Ovarian cancer cells could upregulate PD-L1 in DCs by secreting TGF-β and PGE_2_ into the microenvironment (38), enhancing their inhibition of the T cell immune response. PD-1 inhibitors could restore the capacity of DC, thus enhancing their antitumor effect in ovarian cancer (39). Specific DCs interact with immunosuppressive cells to disturb the antitumor effect. Inducible costimulatory molecule (ICOS) is expressed on the immunosuppressive Treg cells, and pDCs in the ovarian cancer microenvironment activate Treg by expressing ICOS ligand, leading to tumor progression (40).

Some metabolic factors could induce DC dysfunction, including dysfunction of amino acid metabolism and lipid metabolism. The overexpression of indoleamine 2,3-dioxygenase (IDO) in DCs plays an immunosuppressive role. IDO is an essential enzyme in amino acid metabolism, which turns tryptophan into kynurenine. TGF-β released by tumor cells can upregulate the expression of IDO in pDC and the secretion of cytokine CCL22, which recruits Tregs into the tumor microenvironment. IDO-expressing DCs reduce the concentration of tryptophan near Tregs and keep Tregs in an immunosuppressive state *via* tryptophan-induced mTORC-Akt signaling (41). Clinical trials have reported that IDO inhibitors lead to a decreased level of the products of IDO in solid tumors (42), which could be used as a combined agent in DC immunotherapy. Additionally, in response to the endoplasmic reticulum stress induced by the byproducts of lipid peroxidation, the transcription factor XBP1 is activated and this leads to lipid body accumulation in tumor-infiltrating DCs, pushing DCs into a tolerant state in the ovarian cancer microenvironment (43).

Insulin-like growth factor (IGF) also has impacts on the DCs in ovarian cancer. The IGF participate in cell proliferation as well as in protein synthesis and growth through the RAS-ERK and PI3K-AKT pathways (44). DCs treated with IGF fail to mature and secret higher levels of IL-10 as well as TNF-α, which are suppressive immune factors in the ovarian cancer microenvironment (45). The insulin-like growth factor type I receptor (IGF1R) is highly expressed in ovarian cancer and is negatively related to the differentiation of DCs towards cDCs (46). IGF1R inhibitors rebuild the DC-mediated antitumor effect (45), which suggests that the IGF axis may induce DCs to enter a dysfunctional state.

In conclusion, immunosuppressive signals in these aspects lead to a dysfunctional state of the DCs in the ovarian cancer microenvironment. Theoretically, infusion of functional DCs into the body could avoid infiltrating in the tumor microenvironment and instead make direct contact with the T cells in the lymph nodes, which compensates for DC dysfunction state. Based on this, DC vaccines could restore the tumor antigen-presenting ability to elicit antitumor effects.

## Elements of Manufacturing Dendritic Cell Vaccines for Ovarian Cancer

The common routine of DC vaccine manufacturing includes several elements: (1) obtaining human DC developmental potential cells through apheresis; (2) stimulating autologous immature DCs into a mature state *in vitro*, in which process the DCs are usually activated by a cocktail of various cytokines, Toll-like receptors agonists and other activators; and (3) loading the immature DCs with tumor-associated antigens, namely, DCs being cocultured with antigens in the form of peptides, proteins, tumor cell lysates or tumor cells. After these steps, the mature DCs are gathered and vaccinated back to the patients.

Preclinical and clinical studies are exploring various alternatives of each element in the manufacture of DC vaccines to achieve a better efficacy in the treatment of ovarian cancer. These elements are discussed separately below.

### Selecting Appropriate Dendritic Cell Subtypes for Vaccination

The subtypes of autologous DC chosen for vaccine manufacture show various antigen presenting potential, which might affect the efficacy of DC vaccines. In preclinical and clinical studies of the DC vaccines in tumors, DC subtypes selected from peripheral blood cells through apheresis include MoDCs, cDCs and Langerhans cell-type DCs (13, 30). The DC subtypes targeted to improve antitumor immune responses in clinical studies of the vaccines targeting DC *in vivo* and ex vivo are distinct and might be dependent on the cancer types (31). The vaccines targeting DCs *in vivo* do not need apheresis to gather autologous DCs for vaccine manufacturing, and instead, specific antigens targeting receptors on DCs *in vivo* are injected into the body, such as the vaccine CDX-1401 targeting DEC205^+^cDC1s in multiple tumors including ovarian cancer, which contains the DEC205 antibody fused with NY-ESO-1 and a TLR agonist (47).

The vaccines targeting DCs ex vivo are based on peripheral blood cells gathered from apheresis. Among all subtypes, MoDCs are most frequently used for targeting DCs ex vivo, mainly because the count of DCs in peripheral blood cells is not sufficient to produce a vaccine, but the count of monocytes is higher, and the monocytes cultured *in vitro* provide relatively abundant DCs relative to other origins. However, MoDCs show an unsatisfying effect in eliciting CTL responses compared to Langerhans cells in the treatment of melanoma (13, 48). cDCs used for vaccines are also confirmed to superior to MoDCs in eliciting systemic and long-lasting immune responses. Additionally, cDCs could enhance the efficacy of immune check point blockers (49). Flow cytometry and immune bead sorting have made it possible to select specific DC subtypes to induce specific CTL activation. However, there still a lack of evidence to confirm which subtype of DCs is the best choice.

cDC1, cDC2, and pDC are found in ovarian cancer, with a lower rate of both cDC and pDC in the peripheral blood compared with healthy control (33, 35). The ratio of cDC and pDC varies between peripheral blood, ascites and tumor sites. According to The most prominent subsets of DCs is pDC in ascites (50) and tumor sites (34), while cDC is more than pDC in the peripheral blood (35), which suggests that peripheral blood could be a proper resource of the DCs for manufacturing.

However, the counts of cDCs is hardly sufficient for vaccine manufacturing, in most clinical studies on DC vaccines in ovarian cancer, DCs used for vaccine manufacture are MoDCs. Mononuclear cells are isolated from peripheral blood through apheresis and are cultured *in vitro* with GM-CSF and IL-4 for several days. To monitor the cell components of the DC vaccine, the expression of the markers on DCs are analyzed, mainly including CD11c^+^, HLA-DR^+^, HLA-ABC^+^, CD40^+^, CD80^+^, CD83^+^, CD86^+^, and CCR7^+^ (17, 51). Notably, these markers are not sufficient to distinguish MoDCs from other subtypes of DCs, and the final DC vaccine is a mixture of DC and a small fraction of other peripheral blood cells.

To conclude, MoDCs have been most frequently used for manufacturing DC vaccines in the current clinical study on ovarian cancer, and it is unclear if other subtypes of DCs would be more beneficial.

### Loading Tumor-Associated Antigens

To induce DCs to recognize and present specific tumor antigens, several potential methods are tested, including pulsing DCs with tumor-associated antigens, inducing tumor cells and DCs into fusion cells, and mRNA transferring. The tumor-associated antigens are most frequently used in the clinical trials, while the other two are limited to case reports or clinical studies of a small population. The antigens to load DCs determine the specificity of the antitumor effect, production costs, and side effects of the DC vaccine, which makes it an essential step. Among current DC vaccine research on various cancers, immature DCs are loaded with various forms of tumor-associated antigens, including peptides, proteins, and whole tumor lysates. Published studies of different types of DC vaccines in ovarian cancer are listed in **Table 1**.

**Table 1:** The antigen of dendritic cell (DC) vaccines used in the clinicial trials in ovarian cancer.

Antigen loaded	Clinical effect (Survival period)	Published Year
**Her-2/neu or MUC1 peptide**	–	2000 (52)
	In arms 1,2: estimated 3-year PFS: 40% vs 80%; estimated 3-year OS: 80% vs 100%	2012 (53)
	–	2014 (54)
**p53 peptide**	For arms 1/2: median PFS: 4.2 months vs 8.7 months; median OS: 40.8 months vs 29.6 months	2012 (16)
**MHC class I-restricted Wilms tumor 1 (WT1) peptide**	Median OS: 14.5 months	2014 (55)
	Median OS: 13.1 months	2019 (51)
	PFS: 0, 2 months OS: 70, 64 months	2013 (56)
**Neoantigen peptides**	OS since the 1^st^ dose: 15 months	2020 (57)
**Hypochlorous acid (HOCl)-oxidized autologous tumor lysate**	PFS: 1 patient 36 months, 1 patient 44 months	2013 (58)
**Autologous tumor cell lysate**	–	2013 (59)
	Median PFS: 176 days median OS: 198 days	2014 (60)
	In cohort 2, median OS: 11 months; In cohort 3: median OS > 25 months;	2018 (17)
**Keyhole limpet haemocyanin (KLH) and autologous tumor cell lysate**	–	2002 (61)
	Median PFS: 19.2 months; median OS: 43.8 months OS: 64.95 ± 7.62 months	2015 (62)

PFS, progression free survival; OS, overall survival.

For targeting antigens expressed on the ovarian cancer cells, DCs are loaded with one or more peptides/proteins. Proto-oncogene HER-2/neu-derived peptides are used to load DC, such as E75 (epitope recognized by cytotoxic T lymphocytes, amino acids 369-377) (52, 63), GP2 (transmembrane part, amino acids 654-662) (52, 64), and recombinant fusion antigen BA7072, which contains both intracellular and extracellular domain of HER-2/neu (65). Wilms tumor 1 (WT-1) is an intracellular protein that overexpressed in many solid tumors including ovarian cancer, therefore it is targeted by specific cytotoxic T lymphocytes when presented by MHC molecules (66). DC incubated with a MHC class I-restricted modified WT-1 derived peptide [HLA-A*2402-restricted, amino acids 235-243 (51), or HLA-A*0201/0206-restricted (55)] successfully induce WT-1 specific CTL effect, with the assist of a streptococcal primer OK-432 (51). Epithelial mucin 1 (MUC1) is a membrane glycoprotein, which is expressed in 90% of ovarian cancer samples (52, 67, 68). Other peptides for pulsing DCs are selected based on the expression rate in the ovarian cancer, including human telomerase reverse transcriptase (hTERT) (53), pan-DR epitope peptides (PADRE) (53), and p53 peptide (16). Most of the DC vaccines loaded with peptides/proteins have induced peptide/protein-specific IFN-γ secreting T cells proliferation after doses of the vaccines. The overall clinical response rate was approximately 26% (16, 51–53, 55, 65) and the disease stabilization period ranged from several weeks to months, but the clinical responses were limited to stable disease, followed by progressive disease.

To load DCs with antigens that contain a wider epitope rather than single epitope of derivative peptides, fusion peptides have also been tested. For example, MUC1 fusion peptides was conjugated to mannan for synthesizing mannosylated mucin 1 fusion peptide (M-FP) (54, 69). In a phase 2 single-arm study, 21 patients received at least three doses of vaccine DCs loaded with M-FP. Monitored by serum CA-125, two patients had a major response during the study, and the response duration was 57 and 71 weeks, respectively. However, the response rate was only 19%, and the IFN-γ releasing immune response was weak to moderate (54).

To further improve the immune and clinical response rate, tumor cell lysates were considered to be more effective cancer-specific antigens. These whole ovarian cancer cell antigens could be obtained from SKOV3 ovarian cancer cell lines (58), fresh tumor biopsy samples (17, 59), or paraffin block allogenic tumor sample (60). For enhanced immunogenicity of tumor antigens, tumor cells were induced to necrosis by repeating freeze-thaw cycles, or induced to apoptosis by irradiation, as well as oxidized by hypochlorous acid (HOCl). Preclinical study has compared the efficacy of DCs pulsed with different tumor lysates. DC vaccines pulsed with tumor lysates that were prepared through HOCl oxidization followed by freeze-thaw cycles induced higher levels of IFN-γ secreting T cells compared with that prepared through irradiation followed by freeze-thaw cycles, or simply freeze-thaw cycles (58). In clinical trials, the frequency of IFN-γ secreting T cells increased significantly after DC treatment in most of these studies, and the increase of tumor-reactive T cells was associated with clinical benefits (17, 59).

As well as peptide-, protein-, and whole tumor lysate-loaded DCs, the use of mRNA transfected DCs have also been reported as a case report (56, 70). Additionally, tumor-DC fusion cell vaccines in ovarian cancer treatment have been tested (71, 72), but lack sufficient clinical trial data.

In addition to ovarian cancer-associated antigens, nonspecific antigens are also involved in DC vaccine trials. Keyhole limpet hemocyanin (KLH) is a foreign helper protein, which could enhance antitumor immunity by stimulating the IFN-γ production of T cells (73). DC vaccines have been pulsed with KLH and tumor lysate simultaneously; however, some patients developed KLH-specific T cell proliferation but failed to develop tumor antigen-specific T cell proliferation (61). KLH has also been added as a surrogate indicator of a DC vaccine (62), but controlled studies are needed to confirm the association between KLH-specific immune responses and the efficacy of DC vaccines.

### Personalized Dendritic Cell Vaccines Based on Next-Generation Sequencing

Traditionally, DC vaccines are loaded with the tumor-associated antigens mentioned above, but their antitumor effect might be relatively narrow. For the pursuit of a broader antitumor effect, emerging personalized DC vaccines are being developed to target patient-specific neoantigens, namely, tumor-specific antigens that are derived from individual nonsynonymous single nucleotide variations. To validate the individual neoantigens, whole exome sequencing and bioinformatic analysis (e.g., fetchGWI, NETMHC) are combined, complemented or not by high throughput qPCR essays and mass spectrometry (74). To manufacture personalized DC vaccines, DCs are loaded with these candidate individual neoantigens through neoantigen gene-encoding peptides stimulation (75) or mRNA transfection (76, 77).

As a recent cohort study reported, autologous DCs loaded with autologous tumor cell lysate also successfully elicited a personalized neoepitope-specific T cell reaction as predicted (17). In this study, whole-exome sequencing and bioinformatic algorithms were used to predict individual neoantigens, and T cell clones targeted to these neoantigens are amplified after DC treatment. However, the paucity of the tumor sample attained from surgery might be an obstacle, and it is unclear whether the whole tumor lysate would induce DCs to a dysfunctional state as tumor cells do in the microenvironment. A protocol has been published to compare personalized DC vaccines pulsed either with private peptides or with whole tumor lysates (78), which hopefully will provide further evidence about the production of personalized DC vaccines in ovarian cancer.

## Clinical Studies on Dendritic Cell Vaccines in Ovarian Cancer

The safety and efficacy of DC vaccines in the treatment of ovarian cancer has been reported by over 20 studies, including case reports, pilot studies and clinical trials (**Table 2**). These studies either contain multitumors including ovarian cancer (shown with a star mark in **Table 2**), or simply focus on ovarian cancer. Currently, there are 20 registered clinical trials on ClinicalTrials.gov (searched by “ovarian cancer” and “dendritic cell vaccine”). Eleven clinical trials have been completed, three clinical trials are active or recruiting, and two clinical trials are not yet recruiting (**Table 3**). It is important to point out most of the current clinical trials have stagnated before phase II. Recently, a phase-III multicenter, randomized, double-blind, placebo-controlled trial has been registered but is not yet recruiting (NCT03905902), which might provide evidence for the usage of DC vaccines in relapsed platinum-sensitive ovarian cancer patients in the future.

**Table 2:** The clinical trials of DC vaccines in ovarian cancer.

Published Year	Multiple arms of the trial	NO.	Phase of study	Clinical effect	
				Response of DC treatment	Survival period
**2000 (** 52 **)**	Single arm	3*	I/II	2 SD and 1 PD after 3 doses	–
**2012 (** 53 **)**	Arm1 (n=5): DC vaccine; Arm2 (n=6): Cyclophosphamide + DC vaccine	11	I/II	6 NED; 3 recurrence at 6-26 months; 2 recurrence during vaccination	In arms 1,2: estimated 3-year PFS: 40% vs 80%; estimated 3-year OS: 80% vs 100%
**2012 (** 16 **)**	Arm1 (n=14): wild type p53 peptide; Arm2 (n=7): DC vaccines loaded with p53 peptide	21	II	Arm1: 2 NED, 9 RD; Arm2: 2 NED, 5 RD	For arms 1/2: median PFS: 4.2 months vs 8.7 months; median OS: 40.8 months vs 29.6 months
**2014 (** 55 **)**	–	56	retrospective study	1 PR, 7SD, 42 PD, 7 NE	Median OS: 14.5 months
**2014 (** 54 **)**	–	28	II	1 CR, 1 PR, 2 SD, 24 PD	–
**2019 (** 51 **)**	–	3*	I/II	2 SD, 1 PD	Median OS: 13.1 months
**2020**	–	1	Case report	–	OS since the 1^st^ dose: 15 months
**2013 (** 58 **)**	–	5	I	2PD, 2SD, 1 mixed response	PFS: 1 patient 36 months, 1 patient 44 months
**2013 (** 59 **)**	UPCC 11807 (n=6): DC vaccine + bev + cyclophosphamide; UPCC 10808 (n=3): DC vaccine + lymphodepletion + autologous vaccine-primed T cells	9	I	UPCC 11807: 2 PR, 2 SD, 1 NED, 1 PD then PR; UPCC 11808: 1 CR, 1PD, 1 SD	–
**2014 (** 60 **)**	–	7*	II	1 PR, 2 SD, 4 PD	Median PFS: 176 days median OS: 198 days
**2018 (** 17 **)**	Cohort 1 (n=5): DC vaccine; Cohort 2 (n=10): DC vaccine + Bev; Cohort 3 (n=10): DC vaccine + Bev + cyclophosphamide	25	I	Cohort 1: 3 SD, 2 PD; Cohort 2: 1 PR, 4 SD, 5 PD; Cohort 3: 1 PR, 5 SD, 4 PD	In cohort 2, median OS: 11 months; In cohort 3: median OS > 25 months;
**2002 (** 61 **)**	–	6*	I	4 SD for 14-45 weeks; 2 PD after 4-8 doses	–
**2015 (** 62 **)**	14 consecutive IL-2 injections	10	I/II	5 CR, 2 SD, 3 PD	Median PFS: 19.2 months; median OS: 43.8 months OS: 64.95 ± 7.62 months
**2006 (** 71 **)**	–	4*	I/II	2-9 months treatment period	–
**2007 (** 70 **)**	–	1	Case report	PR	–
**2013 (** 56 **)**	–	2	Case report	2 PD	PFS: 0, 2 months OS: 70, 64 months

OC, ovarian cancer; SD, stable disease; PD, progressive disease; RD, recurrent disease; doses, doses of DC vaccinations; NED, no evidence of disease; PFS, progression free survival; OS, overall survival; TILs, tumor infiltrating lymphocytes; Bev, bevacizumab; Treg, regulatory T cells.

*Only the data of ovarian cancer patients are shown here, these clinical studies include more than one type of cancer disease.

**Table 3:** Clinical trials of dendritic cell (DC) vaccine in ovarian cancer registered on ClinicalTrials.gov.

Status (up to 2020.2)	NCT number	Treatment	Number enrolled
**Not yet recruiting**	NCT03735589	DCV, autologous NK cell-like CTLs	18
	NCT03905902	DCV	678
**Active/Recruiting**	NCT00799110	DCV+GM-CSF, DCV+ GM-CSF+ Imiquimod	23
	NCT02033616	DCV+GM-CSF, autologous monocytes+GM-CSF	99
	NCT02111941	DCV	19
	NCT00703105	DCV	36
**Completed**	NCT01617629	DCV	9
	NCT01068509	DCV	63
	NCT00478452	DCV, DCV+ Cyclophosphamide	14
	NCT00683241	DCV	36
	NCT01132014	DCV	67
	NCT01522820	DCV+ Sirolimus	18
	NCT00844506	DCV+ Cyclophosphamide	19
	NCT00648102	DCV	36
	NCT00019084	DCV, DCV+ autologous lymphocytes	70
	NCT00004604	DCV	24
	NCT00027534	DCV	14

DCV, dendritic cell vaccine; GM-CSF, granulocyte-macrophage colony stimulating factor; NK cell, natural killer cell.

### Safety of Dendritic Cell Vaccines

The safety of DC vaccines has drawn great attention for the reason that it might alter the level of immune cells, cytokines, and chemokines *in vivo*. Fortunately, most of the DC vaccines have been well-tolerated by ovarian cancer patients involved in clinical studies. According to the Common Terminology Criteria for Adverse Events, each symptom of side effects is graded by the degree of severity. As listed in **Table 1**, most of the reported side effects are grade 1 or 2, and common ones are local skin reactions, fatigue, pain, flu-like symptoms, myalgia, fever, nausea, and vomiting (17, 51, 53–55, 59–62, 79).

There are several studies reporting serious toxicity of DC vaccines, especially those studies using a combination therapy. In a two-arm, phase II trial of the p53 peptide cancer vaccine and DC vaccine (16), all 21 patients reported a local skin reaction. In the arm that received a combination of DC vaccine loaded with p53 peptide, lymphopenia and fatigue were reported by at least 3 patients. Other reported grade III/IV vaccine-related toxicities are elevated levels of ALT and AST, fever, hypocalcemia, memory loss and rigors. Notably, according to the subgroup analysis in this study, significant toxicity was ascribed to the IL-2 administration. In a phase I trial of DC vaccine in the maintenance therapy for ovarian cancer (17), Tanyi et al. reported that more adverse events emerged in the patients who received a combination of DC vaccine, bevacizumab, and cyclophosphamide. There were grade 3 or 4 toxicities reported by 1 patient each: vasovagal disorder, arthralgia, hip replacement, small intestinal obstruction, anemia, cardiac arrhythmia, and decreased lymphocyte count, and 2 patients reported hypertension. Because these adverse symptoms are also common among ovarian cancer patients following chemotherapy, more evidence is needed to confirm whether these grade 3 or 4 toxicities are related to DC vaccines.

To conclude, DC vaccines are well tolerated in most cases, but a combination therapy of DC vaccines and chemotherapy or immunotherapy should be undertaken only with caution.

### The Efficacy of Dendritic Cell Vaccines in Maintenance Therapy

**Figure 2:**
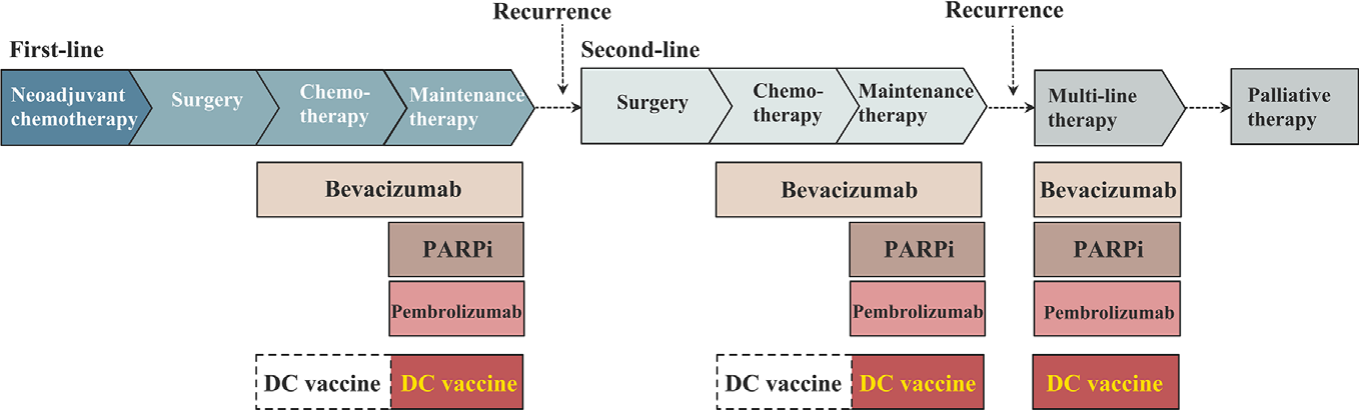
The schema of dendritic cell (DC)-based vaccine in the maintenance treatment in ovarian cancer.

During the past 20 years, DC vaccines for the treatment of ovarian cancer have been most frequently tested during the maintenance therapy (16, 17, 51, 53, 55, 58, 59, 65), with or without other drugs (**Figure 2**). Recurrent ovarian cancer patients are involved in these clinical trials, including chemotherapy-sensitive and chemotherapy-resistant recurrences. Cheryl Lai-Lai Chiang and Lana E. Kandalaft et al. reported a pilot study on a DC vaccine pulsed with HOCl-oxidized tumor lysate in five ovarian cancer patients with recurrence (58). After receiving five doses of DC vaccines injected into the inguinal lymph nodes, two chemotherapy-sensitive recurrent subjects reached stable disease, with progression-free intervals of 36 months and 44 months, respectively. Notably, the PFS responses to DC vaccines of these two patients are longer than the PFS after their previous chemotherapy, which indicates an encouraging effect of elongating remission intervals and a need for further clinical trials with a larger population. Wen Zhang et al. reported a phase I/II study on DC vaccines pulsed with WT1 peptide in three ovarian cancer patients (51). All of these patients were resistant to conventional surgery or chemotherapy, and three chemotherapy-resistant recurrent ovarian cancer patients were involved in this study. Only one patient who had received 10 cycles of chemotherapy responded to the DC vaccines, reaching a state of stable disease and had an improved quality of life. Notwithstanding, it is not fair to conclude that DC vaccines have no effect in chemotherapy-resistant recurrent patients. Compared with the responsive patient in this study, the other two ovarian cancer patients with progressive disease in this study received more cycles of chemotherapy before the trial, namely, they received DC vaccines at a relatively late time. Although the samples involved in these two studies remain limited, it might be more beneficial for ovarian cancer patients to receive DC vaccines at a relatively early time. Due to different study designs, the other clinical studies did not report detailed data or a separate analysis of chemotherapy-sensitive recurrent populations and chemotherapy-resistant recurrent populations, but some of them completed follow-up and survival analysis (**Table 1**).

To evaluate the efficacy of DC vaccines that are received at a relatively early time, namely, before recurrence, Masanori Kobayashi et al. reported a retrospective study on 56 primary ovarian cancer patients who received DC treatment as maintenance therapy following the initial chemotherapy, and 48% of the patients involved in this study continuously received platinum-based chemotherapy during DC vaccination (55). All of the patients were injected with 5–7 doses of DC vaccines, and the median survival times from diagnosis and the first dose were 30.4 months and 14.5 months, respectively. These data show that the first PFS after initial treatment was elongated by DC vaccines. An increased level of tumor-antigen specific T cells were also detected in some subjects, but subjects with immune responses did not obtain a significantly better survival than those without immune responses. However, as a retrospective study with a relatively small sample size, the evidence it provided is not sufficiently strong. Despite this, it provides evidence for the use of DC vaccines as the initial maintenance therapy and confirms that the patient’s nutritional state could affect the DC treatment efficacy, which is useful for the design of future studies.

### Combination Therapy With Dendritic Cell Vaccines

In addition to serving as a mono-drug therapy, DC vaccines are also tested in combination with other therapies, including chemotherapies, targeted therapies, immunotherapies, and nonspecific immune-enhancing agents.

Potential synergistic effects of different chemotherapy drugs in ovarian cancer combined with DC vaccines are distinct (80). Paclitaxel is one of the frequently used drugs in chemotherapy for ovarian cancer. Paclitaxel enhances the maturation of early DCs in mice, and DC precursors exposed to a low dose of paclitaxel express higher levels of CD40, MHC-II molecule, and CD86 in response to antigens (81), suggesting a stronger antigen-presenting function. However, most of the patients involved in DC vaccine trials have already completed primary chemotherapy, and only limited evidence is available to help analyzing the effect of DC treatment and chemotherapy at the same time.

Cyclophosphamide is a nonspecific cell phase agent that prevents cell division by suppressing DNA synthesis, although monotherapy with cyclophosphamide acts poorly against ovarian cancer, and combination therapy with it may strengthen the antitumor effect. The synergistic effects of cyclophosphamide are divergent in two clinical studies. In one study, the administration of cyclophosphamide before DC vaccines did not provide additional survival benefits compared with just DC vaccines (53). However, another study suggested cyclophosphamide might strengthen the effect of DC vaccines. The 2-year overall survival rates of patients with or without cyclophosphamide prior to receiving DC vaccines are 80% and 30%, respectively, and the immune response rate to DC treatment was higher in the cyclophosphamide cohort (17). These two studies differ in many aspects, and one of them is that the latter study also used bevacizumab as a combination drug. Bevacizumab targets vascular endothelial growth factor (VEGF) to suppress tumor angiogenesis while cyclophosphamide decreases Treg as well as MDSC (82), indicating a synergistic antitumor effect with immunotherapy in the tumor microenvironment. Unfortunately, there is no DC vaccines clinical study that provides contrasting groups with or without bevacizumab, which needs further attention.

DC-based therapy might be enhanced by other immunotherapies that have synergistic immune effect, including the immune checkpoint blockers and T cell transfer. It has been demonstrated that immune checkpoint blockers such as anti-PD-1/PD-L1 could theoretically enhance the antitumor effect of DC vaccines (38, 39). However, the expression of PD-L1 on DCs in ovarian cancer patients is moderate compared with on normal ovarian tissues (83), and currently, there is no clinical trial testing the combination of anti-PD-1/PD-L1 and DC vaccines.

Combination therapy with autologous DC and T cells transfer might be beneficial, based on the mechanism that DCs present antigens to T cells. Kandalaft et al. reported a clinical study on DC treatment followed by autologous T cell transfer (59). These T cells were obtained through apheresis after DC treatment and underwent expansion *in vitro*. Seven recurrent advanced-stage ovarian cancer patients received DC vaccines, and three of them that reached PD or PR after DC vaccination were finally enrolled into a T cell infusion group. One of them achieved a partial response (PR) after DC vaccines treatment and later successfully achieved a complete response (CR) after autologous T cell transfer. The second patient who reached a PR after DC vaccination had disease progression, while the third patient had stable disease after DC vaccination and T cell transfer. Moreover, tumor-reactive T cells were detected before T cell transfusion in the peripheral blood of the CR patient but not in the disease progression patient, which suggests that reconstitution of tumor-reactive T cells depends on the immune response to DC vaccines.

Additionally, as a subgroup of T cells, NK cell-like T cells recognize antigens presented by DCs in a CD1c-restricted manner and suppress MDSC in the microenvironment (84), which could help to enhance the efficacy of DC vaccines. A clinical trial of dendritic cell vaccines combined with autologous NK cell-like CTLs for treating ovarian cancer patients (NCT03735589) is carrying out.

Other nonspecific immune-enhancing agents have also been tested as combination agents, including IL-2, IL-12, OK-432, and sirolimus. As Soyoung Baek et al. reported, it was safe to use IL-2 simultaneously with DC vaccines (62). However, another clinical trial reported a combination of DC and IL-2 caused grade 3 or 4 side effects and induced Treg expansion (16). Notably, the administration of IL-2 in these two studies differs in dosage and injection sites, which may account for the divergent results. Recombinant human interleukin-12 (rhIL-12) was used as a combination agent with DC vaccines in various tumors, but limited data reveal the effect of rhIL-12 in clinical trials (71). OK-432 is a streptococcal immunological adjuvant, which is injected simultaneously with DC vaccines but has no significant association with the survival of the patients (51, 55).

### The Administration Scheme of Dendritic Cell Vaccines

There is no consensus on the administration scheme of DC vaccines in the clinical context. DC vaccines tested in ovarian cancer patients are administered intradermally (54, 55, 59, 61), subcutaneously (52, 53, 62, 71), intranodally (17, 58) or intravenously (16, 60). The injection route of vaccination might affect the migration of DCs to lymph nodes, as well as the contact between DCs and T cells. Intranodal vaccination came into the spotlight in recent years. It is reported that far more DCs reach the T-cell areas of the lymph nodes in the melanoma patients administered DC vaccines intranodally, notwithstanding, the immune responses were similar between the two groups (85). But the areas of drainage lymph nodes vary a lot in different cancers, limited data is available to confirm the strengths and weaknesses of different injection routes in the ovarian cancer patients, which left a unrevealed answer for further clinical studies.

The vaccination schemes vary greatly between different studies. In some trials, patients receive a fixed number of DC vaccines at fixed intervals, such as two doses at a 4-week interval (62) or four doses at a 3-week interval (53). In other clinical trials, several doses of DC vaccine are administered to prime the immune response, and residual doses are administered at a longer time interval, for example, five doses every 3 weeks and residual doses per month (17). However, there is no consensus on how to arrange vaccination and examination schedules, which should be carefully considered with when designing clinical trials.

In addition to injection routes, limited evidence is available to confirm the best intervals of injection, the total number of doses to receive, and the administration pathway. Further studies are needed to confirm the administration scheme that most effectively promotes the functional process of DCs *in vivo*.

## Biomarkers to Monitor and Predict the Efficacy of Dendritic Cell Vaccines

Although PFS and OS are considered as the most reliable assessment criteria, survival analysis may take several years to complete. Therefore, sensitive immune markers will be the cornerstone for monitoring and predicting the responses to DC vaccines. Some of the preclinical and clinical studies of DC vaccines have made exploration on two key issues: how to assess the immune responses and how to predict the clinical responses (58, 59, 62), an alteration of immune cells, especially T cells, are in the spotlight of the stage.

### Immune Biomarkers to Predict the Effect of Dendritic Cell Vaccines

Studies on DC vaccines in different types of cancer are exploring biomarkers to predict the clinical response of DC-based treatment. Several types of biomarkers have been reported, including immune cells, cytokines, chemokines, membrane proteins and genes. These immune biomarkers to monitor and predict the effect of DC vaccines in ovarian cancer and other tumors are discussed below.

#### Immune Biomarkers Based on Peripheral Blood Samples

T cell reactivity is the cornerstone of DC vaccine immunoreactivity. In clinical studies of DC vaccines in ovarian cancer patients, alterations of immune cells in the peripheral blood sample after DCs infusion have been demonstrated, including the activation of specific antigen-induced IFN-γ secreting CD8^+^ T cells (58, 59, 62), an increased count of CD4^+^T cells (16) and Th1 polarization (58, 86). These could be regarded as basic indicators, but a monitoring scheme of a comprehensive immune cell profile following DC vaccines has not been established. It should based on both the counts and the functions of immune cells.

The cytokines and chemokines secreted by immune cells are tested as functional indicators. There are significantly increased levels of Th1-polarizing chemokines and cytokines such as IL-12, IL-1Rα, TNF-α after DC vaccine treatment of ovarian cancer patients, while Th2-priming cytokines IL-4, IL-5, and IL13 are at low levels suggesting that DC vaccines elicit a Th-1 antitumor effect (58). A recent clinical study confirmed that DC vaccine-primed CD4^+^T cells to mainly secret TNF-α and IL-2, while CD8^+^T cells to mainly secret IFN-γ and TNF-α (17).

The array of T-cell receptor (TCR) sequences present can be detected by next-generation deep sequencing, namely, the TCR repertoire, which could be used to evaluate the immune response of DC vaccines. According to a cohort study in ovarian cancer patients, there is no overlap of the TCR repertoire between peripheral blood T cells pre- and post- DC vaccines, suggesting that DC vaccines have primed a novel T cell immune response. Moreover, novel T cells manifest high avidity due to high-affinity TCR clones, which benefits DC vaccine-induced antitumor effects (17).

To conclude, the immune markers in the peripheral blood that are altered after DC treatment might be proper indicators of the immune response.

#### Immune Biomarkers in the Ovarian Cancer Microenvironment

Beyond surface markers and the secreting function of T cells in the peripheral blood, characteristics of the ovarian cancer microenvironment might be predictors as well. In the clinical context, a tumor sample could only be obtained during biopsy or surgery, and thus the markers on tumor samples could be utilized to detect infiltration of a sensitive population rather than evaluation indicators of the vaccines.

As a clinical study in glioma patients reported, there was a higher overlap of TCR repertoires between T cells from both peripheral blood and tumor sites, and an increased overlap after DC treatment predicts improved immune and clinical responses to DC vaccines (87).

The molecules on the membrane of T cells can also be taken into consideration. The count of PD-1^+^lymphocytes and the percentage of PD-1^+^CD8^+^T cells are negative prognostic indicators for overall survival and progression-free survival among glioblastoma patients received autologous DCs but not in the control group, suggesting that PD-1^+^T cells infiltrating might be a biomarker for DC treatment (88). In contrast, lower expression of B7-H4, a member of the B7 family, is associated with a better response to DC vaccines in glioblastoma (89). However, neither PD-1^+^CD8^+^T cells nor B7 family molecules has been tested in clinical trials of DC vaccines in ovarian cancer. Further studies are needed to describe the potential of T cells in response to DC vaccination.

One of the limitations in these clinical trials is the lack of the comparison of tumor samples before and after DC treatment, but in the mouse model, such change of ovarian cancer microenvironment has been illustrated, DC vaccination promotes the proliferation of CD4^+^T cells and CD8^+^T cells and decreases the level of MDSCs, Tregs and tumor-associated macrophages (90).

The localization of immune cells infiltrating in tumor sites may has potential impact. Ovarian cancer used to be regarded as “immune desert” due to low level of infiltrating immune cells, but studies have reported the existence of tertiary lymphoid structures (TLSs) in tumor sites, which harbor B cells, T cells and DC-LAMP^+^ dendritic cells (91). The infiltration of DC-LAMP^+^ dendritic cells is associated with better prognosis in ovarian cancer patients (92), but whether DC infusion promotes the build of TLS remains to be explored in the future.

Based on the markers discussed above, an ideal immune response-predicting biomarker should satisfy several points: a strong association with the treatment response or prognosis, a quick examination method to monitor, and a relatively high sensitivity and specificity. However, due to a relatively low response rate to DC vaccines in ovarian cancer compared with other cancers, some biomarkers tested in ovarian cancer but limited progress has been achieved. To explore biomarkers for DC-based treatment responses in ovarian cancer, the basic immune status of the patient, immune characteristics of ovarian cancer and potential drug targets should be taken into consideration. Additional well-designed clinical trials are needed to promote this field forward.

### Association Between Immune Responses and Clinical Responses in Ovarian Cancer

An increasing number of studies have focused on the association between immunoreactivity and the clinical responsiveness of tumor immunotherapy. If specific immune markers can be determined to predict an individual’s immune reactivity to the vaccine, and the long-term clinical benefit of DC vaccines can be assessed by monitoring changes in immune marker levels, it would help to adjust the vaccine dosage and determine treatment endpoints.

The immunoreactivity of DC vaccines in ovarian cancer is mainly described by the alteration of the following immune cells: CD8^+^T cells, CD4^+^T cells, Tregs and NK cells. As a pilot study with 5 recurrent ovarian cancer patients reported, in the patients with a T cell immune response to DC vaccines, the second PFS following DC treatment was longer than the first PFS before the DC vaccine (58). In some studies that have monitored both immune and clinical responses, T cell reactivity is related to clinical benefit, such as a partial tumor response (PR), disease stabilization, and prolonged survival without progression (59, 62), however, these associations between DC-activated T cells and clinical outcome were not stable. A decrease in Tregs after DC treatment could be an immune response indicator, but not a single biomarker to predict clinical response, because a reduction in Tregs has been detected in both stable disease and progressive disease patients (58, 59). Increased NK cell activity after DC treatment was found in ovarian cancer patients in a pilot study. More than half of the enrolled patients presented with increased NK cell activity, but this change was not significantly correlated with clinical prognosis (62). Other immune cells are potential predictors, such as myeloid-derived suppressor cells (MDSCs), which have an immuno-suppressive effect on immunotherapy. Immune responses to DC vaccines are significantly associated with fewer MDSCs (51), which calls for further follow-up to confirm its association with clinical outcomes.

Additionally, serum antibodies IgG and IgM reflect a basic state of the immune environment, which could also bridge the immune and clinical responses of DC vaccines. Patients show weak antibody responses based on IgG and IgM induced by ovarian cancer-specific antigens prior to DC treatment, suggesting a suppressive immune environment in ovarian cancer (54). After DC treatment, the serum IgG and IgM are higher than the baseline in some ovarian cancer patients, indicating a priming immune response *in vivo* (59). However, how long this alteration is maintained and whether it could benefit survival remain to be illustrated.

Currently, studies on DC vaccines differ from each other in aspects of the scale of sampling, vaccine production, and vaccination schemes, which underscores the difficulty in drawing consistent conclusions. Future large-scale cohort studies with a complete follow-up will add additional power to reveal the link between immune responses and clinical responses.

### Timepoints to Monitor Immune Markers

Immunotherapy such as DC vaccines may cause short-term and long-term effects. Immune responses may occur within hours to days or potentially lead to long-term changes in the components of the immune microenvironment. It is essential to capture the alterations of immune reactivity at the correct time, especially in clinical trials where it is unethical to conduct invasive examinations too frequently. In clinical trials of DC vaccines for the treatment of ovarian cancer, there was a significant difference in T-cell reactivity before and after receiving DC (17). The monitoring time points vary from days to weeks (**Table 1**).

To evaluate the immune response to DC vaccines, immune markers should be monitored at least pre- and postvaccination. Examinations at later time points might reflect how long the effect would be maintained. As Christina et al. reported, to evaluate the immune response, examinations were performed at the time of leukocyte apheresis, after the second and fourth doses of vaccine, and at 4, 5, 6, 9, 12 months after the first dose of vaccine. It was observed that antigen-specific effector T cell levels were stable in most patients, but the patients with decreasing T-cell levels showed disease progression (53).

## Future Studies

In the ovarian cancer microenvironment, tumor immuno-suppressive signals induce dendritic cells into a dysfunctional state by affecting the immune function and metabolism of DCs, resulting in difficulty in performing antigen-presenting functions and even promotion of tumor progression. The dendritic cell vaccine provides functional dendritic cells to ovarian cancer patients; thus, it may be a safe and effective immunotherapy for ovarian cancer.

Among the existing preclinical studies and clinical studies, there are huge differences in the preparation process of dendritic cell vaccines, especially the types of tumor-associated antigens used to load dendritic cell vaccines. With the wide application of next-generation sequencing and bioinformatics analysis in various research fields, personalized dendritic cell vaccines have become a hot topic. Because personalized dendritic cell vaccines can activate T cell cloning targets of patient-specific tumor antigens, they can present a more effective antitumor effect. Although the advantages are obvious, there are still some barriers that need to be overcome for personalized vaccines, such as the complicated preparation process, limited amount of tumor samples from surgery, and difficulty in the accurate selection of tumor antigens. Future studies should pay more attention to these challenges.

Clinical trials in ovarian cancer patients have confirmed the safety of dendritic cell vaccines, but the efficacy of dendritic cell vaccines varies with different preparation methods and trial protocols. Most studies have shown that dendritic cell vaccines can prolong tumor progression-free survival, but the effect on overall survival is not significant. The best evidence will need to be provided by prospective cohort studies with large samples in the future. Although some studies have shown a survival benefit from combination therapy with a vaccine containing dendritic cells, adverse reactions are increased, and this approach should be applied with caution. In addition, although no health economics analysis is available, the cost burden of combination therapy is expected to be greater.

Biomarkers to monitor and predict the efficacy of dendritic cell vaccines will significantly push the research field forward, but none of the biomarkers in current ovarian cancer studies perform well. The ideal biomarker should reflect not only the immune response induced by the vaccine but also the prognosis. Immune cells, cytokines, and chemokines are important parts of the immune response, which are candidate markers. The immune character of tumor microenvironment should also be taken into consideration. According to the current studies, no single indicator can meet the requirements, but a combination of biomarkers may be able to reflect the efficacy of dendritic cell vaccines more comprehensively. Future studies should test different marker groups, making full use of the multilevel information available at the gene, protein, and cell level.

To sum up, dendritic cell vaccines have been shown to be effective in immunotherapy for ovarian cancer, but there is still untapped potential that needs to be explored by a combination of new technologies, new cohort studies and new biomarkers.

## Author Contributions

XZ was the major contributor in writing the manuscript and design the figures; TH contributed greatly to build the structure of the review; YL contributed to build the structure of the review and gave suggestions on the manuscript; LC provided suggestions on analyzing the clinical trials on dendritic cells; HL and YW gave suggestions on the structure of the review and the manuscript; HG was the corresponding author of this review, contributed greatly to edit and review the manuscript. All authors contributed to the article and approved the submitted version.

## Funding

Supported by the The Capital's Funds for Health Improvement and Research (No.2020-2-4098).

## Conflict of Interest

The authors declare that the research was conducted in the absence of any commercial or financial relationships that could be construed as a potential conflict of interest.
